# Attitudes, needs, and opportunities for training on musculoskeletal disorder risk reduction in masonry

**DOI:** 10.1186/s12889-025-25011-1

**Published:** 2025-11-04

**Authors:** Tasha C. McFarland, JuHyeong Ryu, Carl Haas, Eihab Abdel-Rahman

**Affiliations:** 1 Department of Systems Design Engineering, University of Waterloo, Waterloo, Canada; 2 Department of Industrial and Management Systems Engineering, West Virginia University, Morgantown, USA; 3 Department of Civil and Environmental Engineering, University of Waterloo, Waterloo, Canada

**Keywords:** Musculoskeletal disorders, Masonry, Construction workers, Apprenticeship, Training, Safety culture, Ergonomics

## Abstract

**Background:**

In many countries, including Canada, employers have a legal obligation to provide training programs to the new workers to reduce the risk of musculoskeletal disorders (MSDs). However, current safety and health training modalities, including those specific to ergonomic and MSD prevention, have shown limited success in promoting safe motions patterns. As workers gain more experience, they develop the knowledge and skills necessary to consistently demonstrate safer and more productive performance in tasks within their areas of expertise compared to novices and apprentices. Training apprentices using expert work strategies is a potential intervention that can reduce MSD risk while balancing productivity needs. By understanding the perspectives of experts in the field, we investigate the specific needs of masonry workers and their employers to improve masons’ safety and health.

**Methods:**

This study conducted qualitative user interviews with eight masonry instructors with more than 20 years of experiences from the Ontario Masonry Training Centre. The eight instructors had an average of 23.9 years of experience as masons with a range between 10- and 43-years. As instructors, they had an average of 6.9 years’ experience with a range between 1.5- and 18-years.

**Results:**

Thematic analysis using template methodology was carried out on the data collected and identified six key themes: knowledge of muscle injury risks and prevention, safety in masonry, physical demands and MSD risk, the impact of physical demands, safety culture and attitudes, and the role of safety in apprentice training. The instructors’ exposure to high physical demands within masonry was a major theme during the interviews. Instructors discussed the high forces, repetition and awkward postures which take a toll on their bodies. Another large theme was about the safety culture and attitudes within the trade. Younger apprentices often think themselves invincible and show less concern towards musculoskeletal safety, whereas the older masons are more concerned.

**Conclusion:**

The findings highlight the need for apprenticeship training programs to include modules on safe lifting practices, ergonomic awareness, and long-term injury prevention. They also emphasize the importance of mentorship from experienced masons, structured rehabilitation support after injuries, and connecting ergonomic practices to productivity outcomes. Instructors’ perspectives provide valuable context to guide the development of ergonomic training systems that are both relevant to masonry work and tailored to the needs of apprentices and their employers.

## Background

Ergonomic hazards and musculoskeletal disorders (MSDs) are significant concern for masonry workers in the construction industry due to the physically demanding nature of their jobs [1, 2]. These hazards, including heavy material handling, repetitive motions, and awkward postures significantly contribute to the high prevalence of MSDs among masons, especially in the lower back and shoulders [3, 4]. According to Hess et al. (2010) [5], block masons manually lift over 200 concrete masonry units (CMUs) per day, with each standard CMU weighing 16.6 kg [6], leading to a total of more than 3,300 kg handled manually each workday. Furthermore, masons spend up to 53% of their work time in a bending posture to pick up and lay down materials at or below knee level, and 38% of their work time in awkward postures [3]. This frequent handling of heavy materials in bent postures exposes masons to severe MSDs and lower back injuries, resulting in the highest overexertion and second-highest back-injury rates among construction subsectors [7].

As new workers in the trade, masonry apprentices are often considered to be in good health in the sense that they have not yet experienced job-related injuries specific to masonry work. However, a recent study reported that approximately 78% of masonry apprentices reported work-related musculoskeletal symptoms (e.g., ache, pain, or discomfort) in at least one body region [8]. Due to competition with their peers and perceived peer pressure to match their seniors’ productivity levels, masons may adopt accumulate productivity-focused but unsafe working methods, particularly at the mid-career level before becoming a journeyperson—certified, skilled tradesperson who completed a formal apprenticeship program and earned a Certificate of Qualification [9, 10]. Therefore, continuous exposure to musculoskeletal risk factors associated with cumulative damage over their career can result in increased rates of MSDs in later years [11]. Despite the high prevalence of musculoskeletal symptoms among early-career masons, MSD risks are often under-prioritized in terms of safety training, and ergonomic principles are often lacking in apprenticeship program [2]. A study by Choi (2012) [12] found that while 91% of construction companies in the U.S. had a written safety program, only 34% had a trade-specific ergonomics program.

The consensus in the literature is that current training modalities (e.g., traditional lecture-based learning, coaching sessions, video review, and biofeedback) have shown limited success in promoting safe movement behavior [13–18]. The main training challenges are the lack of transfer of learning to the work environment or to other untrained tasks [13, 15, 16, 19–23]. Potential reasons for the limited effectiveness of training in changing movement behaviors include that (1) workers tend to resume old habits without reinforcement or refreshment of training; (2) training often occurs in environments and scenarios with optimal conditions and may not consider other barriers and realities of the working environment; (3) task-specific training typically creates improvements for only the task in question; and (4) training will not reduce inherent risks in the job if the physical demands or exposures remain unchanged [13, 24, 25].

Despite these concerns, many countries have implemented health and safety legislation requiring employers to provide education and training on MSD risks [14, 26–28]. This may include instruction on safe work methods, manual material handling (MMH) techniques, mechanical lifting aids, and other interventions that help workers recognize and report MSD hazards [29, 30]. While doubts remain about the effectiveness of current training programs, research has shown that training is the most cost-effective occupational health intervention for MSD risk reduction in 17 World Health Organization (WHO) defined global subregions [31]. Given that training remains an essential step toward reducing MSD risk, and employers have a legal obligation to provide training programs, it is crucial to understand the shortcomings of existing training programs. This understanding can help in developing more effective training initiatives specifically targeting MSDs. By addressing the needs and opportunities within the current training framework, workers’ safety can be enhanced through improved or redesigned training interventions.

As workers gain more experience in their domain, they develop a set of knowledge and skills necessary to carry out their tasks [32–34]. This expertise translates into consistently demonstrating superior performance in tasks within their areas of expertise compared to novices and apprentices [35]. Previous studies, therefore, examined the differences in working techniques of experienced and novice workers by capturing and comparing their work postures and motions, as these are essential components of work strategies [36, 37]. Notably, a consensus has been established that some working postures and movements are associated with MSD risks [38–40]. The use of inertial motion capture (IMC) systems has widely facilitated the collection of a broad range of accurate motion data within the construction industry [41–43] and, indeed, has been used in various applications, ranged from ergonomic assessments to productivity analysis [44–56].

Our recent studies employed IMC systems to conduct a combined biomechanical-productivity analysis on the construction of a standard CMU wall, providing quantitative estimates of masons’ joint loads grouped by different experience levels [9, 10]. We found that journeypersons with more than 20 years of experience adopted previously unidentified techniques to limit the loads applied to their joints while being more productive than their less experienced colleagues. Furthermore, journeypersons were found to adopt a limited and distinctive set of postures and movement patterns different from those of apprentice masons [37, 57]. These findings provide a basis for training apprentice masons based on expert work strategies, which is the focus of the current study, as a potential intervention to reduce MSD risk while balancing productivity needs.

By conducting qualitative user interviews with eight masonry instructors with more than 20 years of experiences from Ontario Masonry Training Centre (OMTC), we aim to understand the specific needs of masonry workers and their employers to improve masons’ safety and health. The primary objective of this study is to gain insights into the safety culture and attitudes within the masonry trades and to assess the current level of knowledge and approach regarding training on MSD risks. Specifically, we aim to (1) gather the insights of instructors and experts on the factors contributing to MSDs in masonry workers, and (2) identify opportunities for developing trainings for apprentices when using equipment and performing tasks.

## Methods

Qualitative interviews were conducted with eight male instructors from the OMTC. Canada Masonry Design Centre (CMDC) reached out to OMTC on our behalf to recruit participants for the interviews. Each participant was interviewed once, with each interview lasting approximately 45 min. The data collection was cross-sectional. The eight instructors had an average of 23.9 years of experience as masons with a range between 10- and 43-years. As instructors, they had an average of 6.9 years’ experience with a range between 1.5- and 18-years. These participants represent the group of OMTC instructors who were accessible and willing to participate through CMDC’s recruitment process. The study does not confirm whether additional instructors were available at the time of recruitment.

Prior to conducting the interviews, a meeting was scheduled with stakeholders from CMDC to refine the interview questions for the target audience. The first interview was conducted with the director of training for OMTC, a previous instructor (able to reprise role as an instructor at any time). This interview was conducted with stakeholders from CMDC present to act as liaisons and to provide additional context for either party as needed. The last 7 interviews were conducted without CMDC present. All interviews were conducted in a semi-structured format and audio recorded with the consent of the interviewees, and the recordings were later transcribed.

This study employed thematic analysis as the primary method for analyzing the interview data. Thematic analysis is a systematic and iterative approach to identifying and organizing patterns of meaning, or themes, in qualitative data [58, 59]. This approach facilitates a deeper understanding of the topic underlying the data set by identifying commonalities [60]. Specific data analysis process included the following steps:

1. Transcribing the Audio-Recorded Interviews: The interviews followed a semi-structured format and were audio recorded with the consent of the interviewees. Post interviews, the audio recordings were transcribed.
2. Initial Coding and Theme Development: To ensure consistency and rigor in the analysis, preliminary coding was completed for all the interview transcripts. This involved systematically identifying and annotating relevant information. Specific codes were assigned to segments of the data that were relevant to the research questions and objectives of this study. Based on these research questions and objectives, a priori themes were developed, including physical demands and MSD risks, experiences with injuries, and injury prevention behaviors (Table 1, left column).
3. Theme Refinement: Emerging themes from the data were clustered with the a priori themes to create an initial template (Table 1, right column). This initial template was then applied to all the data. The template was reviewed and altered as necessary to best fit the data, ensuring that it accurately represented the underlying patterns and themes.
4. Final Template and Thematic Map: After further refinement, a final template was established, which is presented in the results section. This map provided a visual representation of the relationships between themes and subthemes.

**Table 1:** A priori themes and initial template for thematic analysis

A priori Themes		Initial Template		
Safety in Masonry		Safety in Masonry		
•	Physical demands/Musculoskeletal	•	Physical demands/MSD risks	
	disorder (MSD) risks		◦	Age
•	Experiences with injuries	•	Experiences with Injuries	
•	Injury prevention/behaviors		◦	Injury experiences
			◦	Seeking medical help
		•	Safety culture/attitudes	
			◦	Age
			◦	Instructors
			◦	Industry
		•	Learning experiences	
			◦	Experience
			◦	Knowledge sharing
			◦	Instructional courses
		•	Role of safety in courses	
			◦	As apprentice
			◦	Current curriculum
			◦	Informal teaching
			◦	Advice
		•	Risk modifiers	
			◦	Anthropometrics
			◦	Age
			◦	Causes of injury
			◦	Fitness/conditioning
		•	Safety Behaviors	
			◦	Stretching/warm-up
			◦	Technique
			◦	Equipment
			◦	Other

**Fig. 1:**
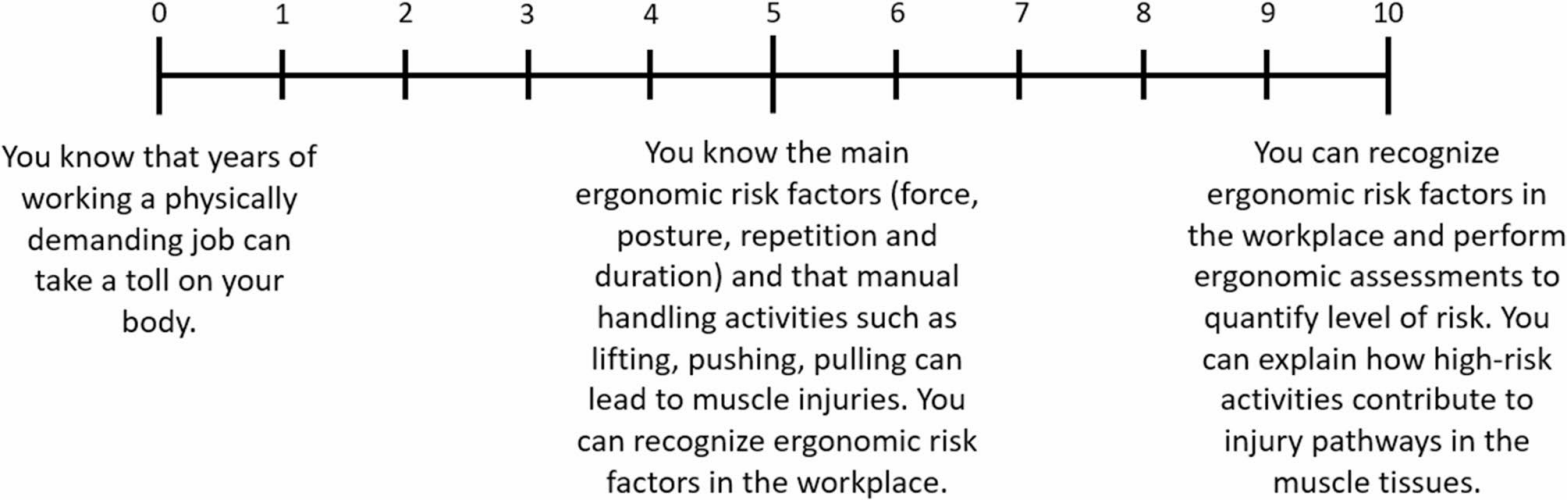
Scale used to rate knowledge of muscle injury risks in masonry

**Fig. 2:**
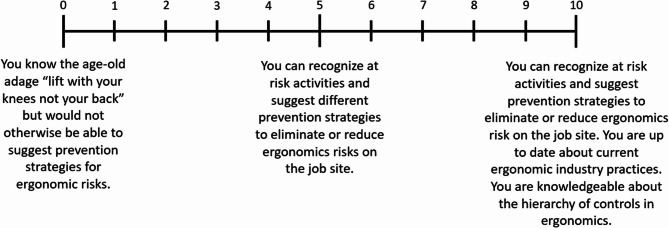
Scale used to rate knowledge of prevention strategies to reduce muscle injuries

In addition to thematic coding, instructors were asked to rate both their own knowledge and that of their students (1st, 2nd, and 3rd year apprentices) on muscle injury risks and prevention strategies using 0–10 scales. These scales are illustrated in Figs. 1 and 2, which outline the criteria for each rating level. This approach provided a structured way for instructors to assess and compare knowledge levels across apprenticeship stages, supplementing the qualitative interview data with a semi-quantitative perspective.

## Results

**Fig. 3:**
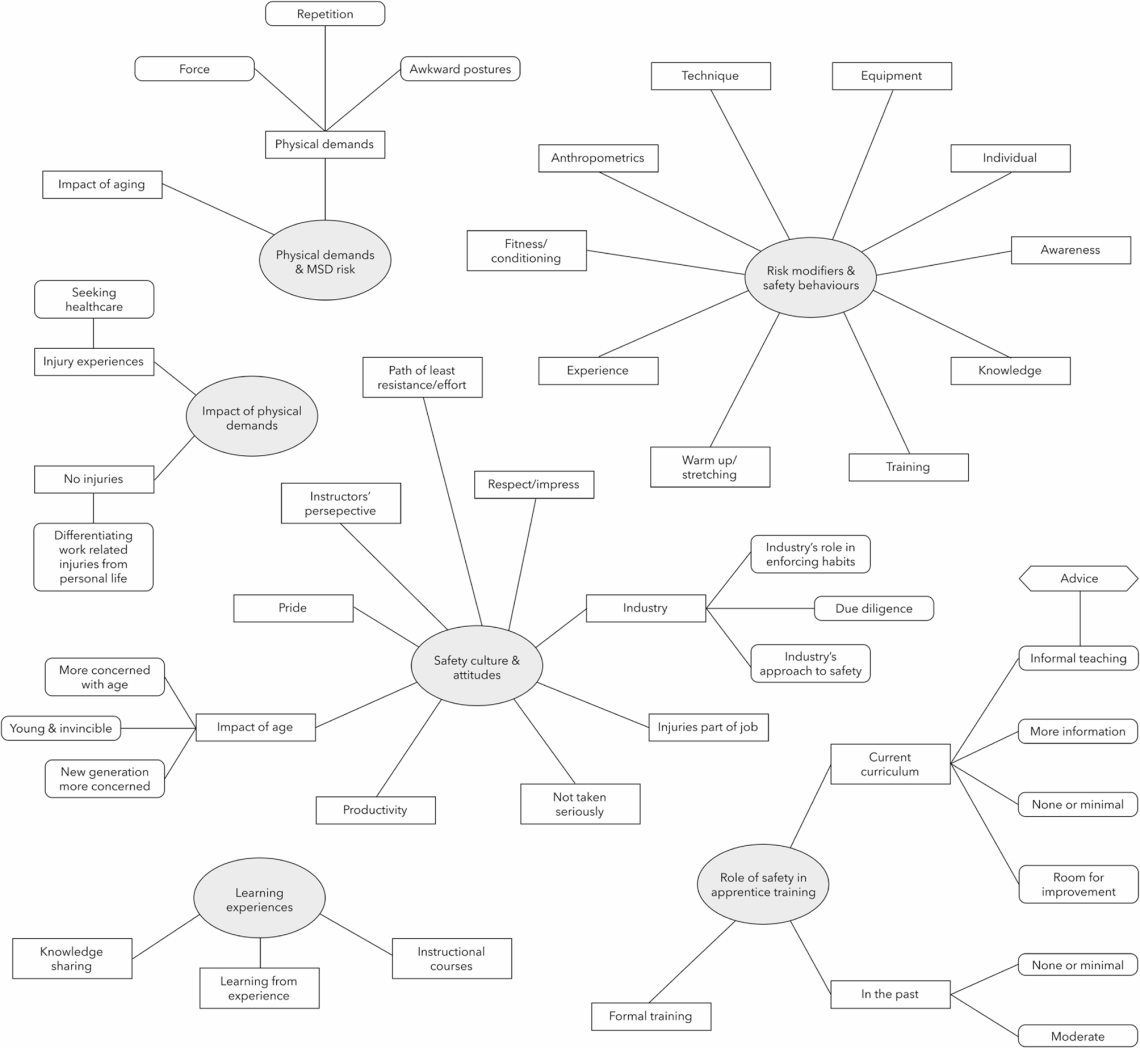
Thematic analysis map of safety in masonry

On the topic of ergonomics in masonry, the eight instructors described their own experiences in the trade, emphasizing physical demands, injuries, safety culture, learning experiences, the role of safety, and risk modifiers (Fig. 3). The thematic analysis map outlines the main themes identified in the interviews regarding ergonomic knowledge and attitude in masonry. The main themes include: (1) Physical demand and MSD risk, (2) Impact of physical demands, (3) Safety culture and attitudes, (4) Learning experiences, (5) Role of safety in apprentice training, and (6) Risk modifiers and safety behaviors. Within these themes, several subthemes were also identified, reflecting more specific aspects of safety practices and experiences reported by instructors. For example, under the Learning Experiences theme, three subthemes were identified: (a) Knowledge sharing, highlighting the informal exchange of practical insights and techniques among masons; (b) Learning from experience, reflecting how direct encounters with muscle injury risks shaped masons’ understanding and practices; and (c) Instructional courses, referring to the structured training sessions focused on injury prevention and safety. Together, these themes and subthemes illustrate the overall culture, attitudes, behaviors, and experiences of apprentices and masons related to muscle injury risks and prevention. Each theme and subtheme is elaborated further in the following sections.

### Knowledge of muscle injury risks and prevention

**Fig. 4:**
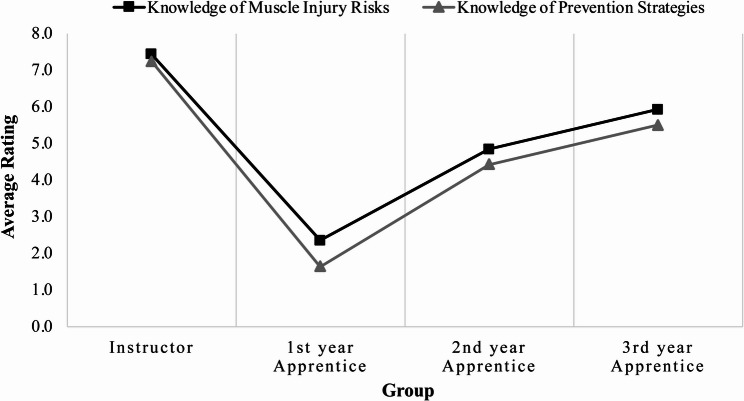
Instructor and apprentice knowledge of muscle injury risks and prevention strategies in masonry

On average, instructors rated themselves similarly for both muscle injury risks and prevention, but for apprentices, they rated their knowledge of prevention strategies slightly lower (Fig. 4). Instructors had the most knowledge of the experience groups with an average rating of 7.3–7.4 whereas the 1 st year apprentices had the least (1.6–2.4). The apprentices’ perceived knowledge increased as they gained experience. The greatest improvement occurred between the 1 st and 2nd years, with increases from 1.6 to 4.3 in prevention strategies and from 2.4 to 4.94 in muscle injury prevention, representing increases of 106.1% and 169.6%, respectively. The increase between the 2nd and 3rd years was smaller, at 22.1% to 24.1%. While 3rd-year apprentices achieved the highest knowledge ratings among the apprentice groups, their perceived knowledge still remained approximately 20.3–24.1% lower than that of the instructors.

### Physical demands and MSD risk

Strenuous physical demands were a major theme within the instructors’ relationships to their jobs and to the topic of musculoskeletal risk and prevention. The instructors highlighted the demands placed on their body every day, and the impact of aging on their ability to withstand those demands. High forces, repetition, awkward postures, and lack of rest were highlighted in the instructors’ accounts as major demands within the trade:

*Like it’s there’s a lot of lifting*,* bending*,* crouching*,* working on your knees… you know it’s very hard on the joints that’s just all there is to it*,* right.*

*Its just a lot of repetitiveness*,* understandably enough we’re having our trowel in our hand other than lunch and break… it’s 40 to 50 h a week*,* right. So. Your hand is always clenched tight*,* so*,* and then of course there’s like laying --laying blocks is really more the demand of it. Bricks is one thing*,* its nice and light*,* it’s in your hand*,* but when you’re lifting a 50–60 pound block over and over and over and over and over again. It definitely beats up your body quite quickly*,* right.*

*If you lay block for an entire day*,* you likely move a few 1000 pounds of mortar over your trowel. So just with one forearm*,* you’re moving hundreds or thousands of pounds.*

Many of the demands were emphasized as part of the job. That the physical demands were the reality of the trade, and that while prevention is important, elimination is not feasible or realistic:

*These materials that you’re lifting are never gonna change. The dimensions are always going to be the same and the weight will always be there.*

Instructors emphasized the toll that these demands can place on masons’ bodies over the long term:

*I’m a firm believer no matter how fit you are*,* if you’re doing masonry all your life*,* you’re punishing your body plain and simple. So*,* about the time you’re 60–65 years old*,* if you haven’t figured out something else to make a living… Like if you been a journeyperson*,* let’s say*,* which some guys are*,* that’s fine*,* they want to lay brick and block all their life*,* get their check and go home… there’s gonna be permanent damage there… there’s 100%*,* just no two ways.*

Several of the masons touched on how aging reduces their physical capabilities to keep up with the physical demands of the job:

*This trade is no joke*,* I mean*,* it’s– and especially if you want to stay in it for 10*,* 20*,* 30 years. It’s you know it’s a humbling trade*,* I’ll tell you. It doesn’t get any easier*,* it only gets harder because your body of course requires more of itself as the years go on*,* and you’ve got less of it*,* right*,* less muscle*,* less patience*,* less everything. […] Five years ago I was not even thinking of teaching*,* I was just laying block*,* doing my thing and hey I could do this forever*,* right*,* and then all of a sudden*,* just something changes in you*,* something changes in your body*,* and it’s very scary*,* right.*

Even one of the youngest instructors at OMTC, noticed the effects of aging on his body with respect to the job:

*As I age*,* I do notice every action is just mildly more difficult.*

### Impact of physical demands

Most of the masons mentioned that the physical demands had taken a toll on them and resulted in some minor wear or tear; however, only a few mentioned having some experience with injuries. Of those that had injuries, the severity of their experiences varied. Several mentioned their personal experiences with being injured due to the job demands:

*So that that little window of my life led to a wrist injury*,* but I quickly*,* you know*,* I saw some help but I got some advice and guidance and then I kind of changed my view and adjusted my approach in sort of– so I dealt with that injury at that time. So*,* it wasn’t like years and years of problems*,* it was like maybe six months to a year*,* where I kind of got through my stubbornness and I figured it out.*

*I mean I’ve had back issues off and on throughout my career.*

*I felt something weird one day like this is like my first or second year as an apprentice and I was moving a wheelbarrow and I stepped on something and I had a little pain in the lower back and I wasn’t sure what it was*,* anyways*,* I left it alone and it didn’t really bother me and then I’d say 4–5 years later I was lifting some bricks out of the back of the truck and I was in a weird position*,* I forgot to do the usual warm-up routine and I felt like I pulled something*.

*Shoulders for sure. My right arm for sure. It goes without saying… three maybe four days in a week*,* my arm in the middle of the night is completely numb.*

For those who had experienced an injury, it was usually severe enough to seek medical treatment. Instructors sought the help of a variety of healthcare professionals including chiropractors, osteopaths, physiotherapists, and doctors. After receiving proper treatment, masons were able to recover and go back to their job:

*But like I said low and behold I ended up going to physiotherapy and the lady there had this --basically described to me that the scar tissue over the tear I had was basically fusing my shoulder in an incorrect way based on the injury I had. So she literally bent this thing backwards and forwards and she goes I’m sorry I know you like --you don’t want to show up here anymore ‘cause you’re in pain but she goes I have to do this. I have to bring your shoulder back to the way it was. And honest to God I can’t thank her enough because my shoulder feels 100% brand new again.*

Only some mentioned persisting injuries post treatment. On the other hand, several masons have mentioned that they have never had a major injury over the course of their career:

*I’ve never been hurt. I’ve never been off work… yeah feeling pretty strong still. Yeah*,* I just don’t know*,* as I’m getting up in age it’s maybe the lower back’s starting to feel it*,* but… knees*,* in my elbows but… nothing that’s hindering me from doing my job.*

To deal with some of these physical demands, some instructors mentioned substance use. One instructor mentioned relying on drugs from their doctor to deal with the muscle pain while another brought up the problem of substance abuse in the construction trades:

*It’s in construction*,* you know*,* any real trade*,* but go any trade to the next and there’s a lot of substance abuse*,* well often because people are going to work sore and then you know*,* the first one’s free and the trades are hurting*,* and they’re physically… not just bricklaying.*

When reflecting on how the physical demands of the job have impacted them, several masons also mentioned that it was hard to distinguish which impacts on their body were work related and which were related to recreational sports activity outside of work or aging:

*I mean I don’t know where you cut the line between aging and what your work is doing to you*,* right. It’s hard in my personal life too I also… I’ve been playing soccer since I’ve --I was four and so those physical activities are definitely not helping either. So*,* it’s really hard to know what part of*,* you know*,* my physical being has to do with work and where it has to do with soccer and everything in the middle. Because I’m also an active individual*,* right.*

### Safety culture and attitudes

Safety culture and attitudes towards safety was another theme in the data. All the attitudes described were interpreted through the perspective of the instructors themselves, including their own experiences and attitudes as well as their perceptions of the apprentices’ attitudes and the industry’s attitudes towards safety and ergonomics.

One of the most common aspects brought up by the instructors was the impact of age on safety (75%). Most of the instructors said that younger apprentices were not as concerned about the impact of the physical demands on their bodies. Young apprentices often tend to believe they are invincible and approach tasks with this attitude:

*Everyone is young and that so*,* you know*,* you’re gonna beat your body up because you’re invincible.*

*When I was young and 21…22 and getting into masonry the last thing I was thinking about is how to stretch and I mean you’re –you’re fit*,* like Superman*,* right. So*,* end of the day you’re not really too overly cautious about it.*

On the other hand, the older masons are more concerned about the impact on their body, and take the steps to prevent injuries:

*It’s actually kind of funny ‘cause we were talking and guys were just giving stories back and forth*,* and they were saying you know on this commercial site where you get into an elevator*,* the young guys are getting in the elevator and going all the way up and you see the older boys are walking the stairs all the way up and of course young guys like why would you want to take the stairs and they’re like this is how we warm up our muscles*,* right?*

It is only as the younger apprentices age, and by extension gain more experience, that they become more concerned about the muscular strain of the trade (62.5%):

*Yeah*,* for sure*,* 100%*,* that changes. Because either (a) they get injured or (b) they see someone that gets injured or 3) or (c) they get to a point their life where they start to realize that they’re not gonna be young and healthy forever so now their focus switches to health which coincides with their work environment.*

Conversely, some of the other instructors said that the incoming apprentices in the current generation are often more concerned about the physical demands of the trade (25%), compared to apprentices in the past:

*Yes. More and more of them [are concerned about the physical demands] if I’m being honest. It was not that way when I came in a decade ago. Again*,* it was still kind of a– I don’t want to say rougher environment*,* but in general I feel that trades are changing really fast for the better*,* which is a great thing. […] I’m gonna say the vast majority of them are concerned. When I do info seminars where we talk to students at high schools*,* where people interested in our pre apprenticeship programs or entry level programs*,* it’s one of the most common questions.*

In the masonry trade, income is based upon work performance, which creates a pressure to maximize productivity (50%). This emphasis on productivity can come at the cost of their own physical condition by taking on too much, cutting corners or not taking the time to think things through:

*So much is judged on your output and for myself my income is judged on my output so I do pull sometimes more on myself than probably should and until I get to that point where I feel sore*,* I probably go past where I should physically. And I do not monitor it until it flares up*,* which isn’t something I’m proud of and it’s something I should work on but it’s --that is the truth.*

Around half of the instructors mentioned that apprentices often have an indifferent attitude towards safety, and that they find it hard to get through to the apprentices:

*On a pack of cigarettes*,* they have these terrible looking things about*,* you know*,* bad lungs and this and that. All these pictures but as soon as they get used to the pictures*,* it doesn’t mean anything to them*,* they just buy the cigarettes anyways and the safety is the same thing. […] I think until you can convince somebody between their ears that safety is some value to them*,* I think*,* until you can convince them between their ears*,* that safety is of value to them… I think you could teach it as much as you want*,* all day long whatever*,* I don’t think it’ll make much of an impact and I’ve seen this so many times*,* firsthand.*

There is also an overall attitude within the trade that injuries are just part of the job, which was expressed by several instructors:

*It’s part of the job*,* you’re gonna get hurt. The lifting’s always the same*,* you always got to lift.*

Not only did instructors reflect on their own and other masons’ attitudes, but they also highlighted the impact of the industry on the overall culture and its contribution to safety attitudes within the trade. While the interview with instructors focused on the ergonomics within the apprentices’ skill training classes, they emphasized that the industry plays a role in not only training proper habits but also setting expectations for workers. While ingraining good habits is critical in trade school, it’s only a part of the big picture:

*A lot of that too is we only get them in trade school for a little bit then the industry has to do the rest of the work. Whether they’re learning it from us or they’re learning good or bad habits from the industry. If you learn something one way*,* but then*,* you know*,* when you get into the industry and it’s… the stresses and demands*,* you know*,* you might… that’s where you kind of that’s where you let your hair down and make those mistakes. I know contractors want to make money*,* but do they want to lose time on injuries. I’m not asking about steps… maybe 100 less blocks a day or something*,* you know*,* but the industry plays a role in this too and what they set as expectations for people.*

Nevertheless, several of the instructors commended the change in approach to safety within the last decade. Overall safety is being taken more seriously within the industry and the culture towards injuries is starting to shift to the point where getting injured on the job isn’t just accepted:

*Nobody used to talk about it. It was one of those things*,* that years ago in construction*,* you just had to --your knees or your back will go*,* that’s how it is and then you have to find something new to do. It wasn’t looked at as a long-term career by most people*,* so nobody --and that was the trade-off*,* you make a decent wage*,* but your body may breakdown eventually*,* just like many other things. And that’s no longer acceptable*,* which is good*,* it shouldn’t be*,* but I get because of that --the shift is changing and the next generation cares a lot more about that.*

This stance is a bit at odds with other attitudes in the industry, where getting injured is viewed as part of the job. However, perhaps this reflects that the overall culture is still shifting and has farther yet to go. In line with a cultural shift towards safety, the technology implemented on job sites reflects this higher standard for preventing injury:

*In my 43 years*,* I’ll tell you*,* there’s a lot more equipment that lifts things*,* whether it be you*,* or you and your materials*,* elevating platforms*,* like climbers on the side of the… like elevating scaffold basically*,* keeping you at the perfect working height all the time. That’s gone a long*,* long way*,* you know*,* machines that can telescope and put materials higher on things and it just eliminates all the back-breaking work. Really*,* I can attribute*,* I can see it too*,* the fact that there’s more women getting into the masonry trade and that’s because there is left less lifting involved to certain extent. […] I’ve worked on parliament hill for six months and it doesn’t matter how old you are*,* whether you’re 7 years old or 77 years old*,* you can lift stones…you know… half as big as your car because there’s chainfalls on site. It’s stuff like this… they’ve eliminated so many of those hazards of lifting… and devices to lift things into place*,* pipes to slide them on. Yeah*,* the workplace has definitely gotten better.*

On the other hand, one instructor had a more jaded perception of the industry’s role in safety and safety training. He personally felt that large companies in the industry treated safety and ergonomics training as just fulfilling their due diligence:

*I’ve talked to too many people*,* very high up the ladder on safety*,* and a lot of large companies*,* I mean*,* they just build it into the price*,* you know*,* it’s 10% of a job and we’ll make sure we deliver all these courses and do all these things so it looks like we’re doing our due diligence. But we already know that they’re not likely… that the safety… the incidences are going to stay the same rate. That’s not their concern… their concern is that we’ve delivered the course*,* we’ve done our due diligence and you know*,* if something happens*,* they can’t be blamed on us. It’s kind of a sad statement but that’s what I see it’s been reduced to.*

Instructors mentioned that apprentices have a lot of respect for the individuals who have trained them and want to get that respect in return or want to impress them. Sometimes this culture of trying to gain respect or trying to impress can result in apprentices pushing past their limits:

*I think that also they want to earn their badges in regard to getting respect from the elders and in order to do that*,* they have to work a little bit harder than the average joe. And sometimes when people push their bodies and their minds to the limits*,* they make mistakes as well.*

Similarly, masons take a lot of pride in their work and in their abilities and this culture can also lead to masons pushing past their limits:

*Much of my pain is self inflicted. It’s I don’t know if it’s a pride thing or what --it’s definitely a pride thing. I like to work hard. I like to try and put out the most I can. And not super proud of it but I guess I’m not ashamed of it*,* like many masons I like to attempt to put in more than the masons around me.*

Instructors also mention that masons will take the path of least resistance or effort just to get the job done, whether that be lifting in the way that they’re used to, or is the fastest, or skipping out on getting help:

*It’s the last stone that’s got to go in*,* I should get some help… I think I can do it*,* that type of thing and then*,* have a little bit of regret later on or whatever.*

Despite various attitudes within the industry that could contribute to injury risk, all instructors thought that safety and ergonomics were important. Many instructors emphasized safety within their classes through informal means such as advice or other messaging they share with their students. Typically, instructors share their own unique perspective on the importance of ergonomics and safety framed through the lens of their own experiences:

*I do mention it*,* because it– probably because again I am– I am already our youngest instructor to my knowledge*,* there’s a few others around my age but it has impacted myself and then other people that I know… so I do try and mention it when I teach just so they’re aware of it. […] I try --my exact line to them in the course is that if you go through your career and you put your time in*,* so you can retire and then by the time you get there you can’t walk because you destroyed yourself*,* what was the point? Because that’s how I look at it. I use the same speech for safety gear*,* like safety glasses and earmuffs and things like this.*

### Learning experiences

A critical difference between the expert masons and the apprentice masons is their implicit and explicit knowledge. As such, identifying how expert masons learn and gain their skills is essential. A central goal to our research is using these findings to transfer knowledge more effectively from expert to apprentice masons to make the training process more efficient.

One of the main ways that the instructors learned about muscle injury risks and prevention, was through the sharing of knowledge (75%) between other masons:

*Which again goes to stuff you learn from other masons*,* teaching you stuff and it’s just something’s quicker and faster and easier on your body […] Even just the way a bricklayer taught me how to spread my mortar with just the turning of the hand as opposed to moving your entire shoulder and body around thing.*

Another common way masons learn about muscle injury risks and prevention strategies is through experience (62.5%):

*The blocks don’t ever get lighter*,* so yes*,* the blocks are the blocks and that’s all they’re gonna be. And scaffolds are going to be scaffold and heights are gonna be heights*,* and so none of those components will ever change*,* right. It’s only as you get into the trade*,* year after year*,* you’ll learn the tricks of the trade… you’ll learn how to do things much more efficiently and try and save your body while doing it*,* right.*

*I’ve done a lot of restoration work as well… taking large stones out of walls and I think I’ve become more adept at seeing the hazards and how-to kind of nip them in the butt there before they happen. [Referring to how he learned to see the hazards] Oh 90% is experience for sure.*

*Because by that point if you haven’t identified the motions that can wound you or that*,* you likely just haven’t been on a job site very much.*

Many of the masons thought that apprentices were at greater risk for injuries, for multiple reasons, one of which was lack of experience. Several commented that if the apprentices didn’t learn quickly, they’d hurt themselves:

*Again*,* if they’re still making the same mistakes and possibly by third year*,* they’ve hurt themselves with this*,* so they have an understanding.*

*You should pick it up fairly quickly like if you don’t figure out how to pick up the block very quickly you won’t be without hurting yourself. You won’t be in the trade very long.*

Only one instructor mentioned learning from a course, when external consultants were brought into the classroom to teach the apprentices about lifting techniques. While he was an instructor at the time, he also learned from the demonstrations that were given to the students.

### Role of safety in apprentice training

Another theme was the role of safety, particularly muscle injury risk and prevention, in apprentice training throughout the instructors’ careers: in the past, as apprentices themselves, and in the current curriculum as instructors. Referring to their own experiences as apprentices, many instructors (62.5%) mentioned that there was little or minimal focus on ergonomics during their skills training courses:

*Yeah*,* there was very little information when I was an apprentice*,* there was very little information about that sort of thing*,* you know. I’d say almost zero.*

*I mean when in school we were told so you know obviously stretch before you start working and you know stuff along those lines*,* but certainly to go into depths… not much to be honest with you.*

Only a few instructors (37.5%) said that there was a moderate amount of information or focus given to muscle injury risks and prevention when they were an apprentice:

*It was one that was warming up*,* right*,* it was warming up the body in the morning*,* so we discussed that*,* and the second part was learning how to lift heavy objects without pulling muscles… so proper stance*,* which way to move your body while carrying weight…um what else*,* yeah in regards to actually doing the job there was… ways of moving your body with heavy material without creating injury*,* the way you walk*,* the way you position yourself and lift something up on material plank*,* etc. etc. so we did discuss that*,* yeah.*

The director of training mentioned that in the current curriculum, the students have more access to information about ergonomics, and that overall safety has a greater emphasis:

*Well*,* it’s mostly through any of our health and safety courses that we do*,* that would be the majority of where they’re getting the type of information that I didn’t get when I was an apprentice. So not all of it would apply to… specifically what you’re talking about*,* but in general terms*,* safety is stressed a lot more and on the job site it’s monitored a lot more*,* in terms of what people do and don’t do and so on.*

However, when it came to the other instructors, many said that they don’t have anything, or very little in the curriculum that covers muscle injury and prevention:

*Currently we don’t have a specific curriculum about injuries of the body.*

*Because like I say*,* in the last class I had*,* there was never a time period where we took two or three hours and I showed them*,* how to*,* you know*,* bend and lift*,* I mean you just don’t do it.*

*There’s a section prior to tools and equipment*,* and they discuss*,* you know*,* eyeglasses*,* personal protective equipment*,* basically*,* and in there*,* there’s a section on the ergonomics of trowel size*,* stuff like that*,* but that’s about the extent of it.*

Overall, the instructors indicated that the current curriculum is lacking and there’s room for improvement:

*I don’t think we hit very hard at all. The Ontario Training Masonry Center.*

*The actual physical activity and working out and those types of things*,* probably… there’s probably still a good… still lots of room for improvement*,* let’s put it that way.*

Conversely, while there was no material in the curriculum address muscle injuries and risks, most of the instructors still touched on the topic during their classes through informal teaching methods (62.5%), such as demonstrating techniques or correcting the apprentices’ techniques:

*When we teach how to spread mortar and pick up block*,* I teach what works for me and I’ve never had a problem*,* you know. So*,* I must be doing something right.*

*When I see people picking mortar up off their board with their trowel and they flick in the air for the suction I try to make my… I remind… I stop them and I tell them as many times as I can see them doing it*,* that the more you do that the more you’re going to be pulling on that arm and I show them maybe just a light tap on the board instead and hopefully they understand that and then they go forward in the rest of level 1 not flicking in the air*,* maybe down the rest of their careers I might have saved their elbows.*

Instructors also focus on muscle injury prevention by giving advice (75%) and stressing the potential long-term impacts of the trade:

*I just give as much information as I can about what they could be doing to help themselves or making them aware that these injuries will exist and do exist but again*,* as far as curriculum goes I don’t have a… I don’t think we’ve been given a chapter*,* you know*.

*So now it’s a matter of taking that advice from a veteran like me that’s been doing it for almost two decades and saying listen like you have to stretch you*,* you have to bend*,* you have to because otherwise you will pull a muscle and tear it.*

*I mean all the anecdotes I have*,* it’s that you know*,* I used to jump off the scaffold at the top plank too and now it’s every step down*,* you know*,* ‘cause that… because people told me that*,* I didn’t believe them but then eventually it catches up you. You know*,* just try to sound as crotchety as a bricklayer as I can and so they get that it’s going to affect them.*

All the instructors thought that material on muscle injury risks and prevention should be incorporated into the curriculum formally:

*100%. 100%. Whether it’s a few hours presentation or whether it’s a day thing… like I don’t wanna exaggerate it*,* but certainly like I say*,* the better off people are with anything they do in life*,* information is key … we put so much emphasis on working at heights*,* we put so much emphasis on… WHMIS and with everything else… especially in the masonry field we should be putting emphasis*,* I feel*,* on your body*,* your muscles*,* you know*,* how they react.*

### Risk modifiers and safety behaviors

Another major theme throughout the interviews were risk modifiers and safety Behaviors. Risk modifiers are attributes that might increase or decrease an individuals’ susceptibility to musculoskeletal injury. Similarly, safety Behaviors are behaviors that masons would engage in to prevent musculoskeletal injuries. Analysis of these attributes and Behaviors between and among apprentices and experienced masons revealed circumstances that might contribute to higher or lower risk of injury.

Experience was noted as one of the main factors affecting risk of injury (87.5%). This is likely correlated to revelation that one of the primary ways, masons learn about muscle injury risks and prevention is through personal on the job experience. As previously mentioned, instructors thought apprentices were at greater risk for injury partly because of their lack of experience in the trade:

*They’re inexperienced*,* anyone inexperienced in any field is at risk to make a mistake*,* unfortunately in construction*,* mistakes can lead to injury.*

*I’d probably jump to the conclusion that the injuries are going to happen early on*,* in my view*,* ‘cause […] they don’t know their bodies as well as they will in three or four or five years.*

Fitness and work conditioning were other factors associated with injury risk (75%). Instructors indicated that early on, not being conditioned to the physical demands in the trade could increase the risk of injury:

*And they’re just more open and more susceptible and parts of their bodies that haven’t been worked in the way that it will be in our trade. Certain muscle groups that will now be exercised that perhaps were not exercised.*

On the other hand, instructors said that a level of personal fitness and conditioning that meets the job demands is protective and reduces injury risk:

*Staying physically active*,* staying physically fit is definitely imperative when it comes to masonry.*

*I use the same set of muscles over and over and over*,* repetitively […] I guess I’m just used to the way I work […] the only time there’d be a problem is if you do something out of the ordinary*,* something you’re not doing every day.*

Several instructors mentioned that anthropometrics (62.5%), such as body size and height, impacted masons’ capabilities:

*I think a lot of that has to do*,* there is --I hate to say this but there is a certain point where your physical size and your body type has a role to play in that. I have --again I have acquaintances who are in this trade and they are fairly large individuals like they’re large frames. Like I feel they can stand up to the strains of laying concrete block on a daily basis*,* where I can do it for a few months*,* with my size --like I’m not small. I’m not big. I find that actually a lot of masons are around my size. Like I think I’m about 5’11”*,* 180 pounds. Continually picking up a block again and again and again and again and again that weighs 65 pounds and then lifting it over a string to put it in a wall. I feel it at certain points depending on where the height is etc.*,* kinda just like my extension*,* my range of motion. I feel it in my lower back. Just ‘cause I do not feel I have the weight --the counterweight that block*,* because I’m just not that large.*

Technique also factored into susceptibility to injury (62.5%):

*I think a lot of that came down to not doing things that I knew would contribute to that. So*,* the way that… you know*,* where I lifted objects from and how I move my body… lifting*,* you know*,* using the knee*,* keeping things close to my body*,* rather than away from my body*,* and it’s those times when that wasn’t possible that [injuries/pain] popped up again.*

The importance of warming up or stretching were also emphasized by a lot of instructors to reduce injury risk on the job site (50%):

*I definitely let my younger and older students know*,* especially to be honest with you*,* the older ones… stretching is a known fact that if you don’t stretch*,* you can easily pull something*,* tear something*,* and then you’re really in trouble right. So yeah*,* I definitely promote stretching before you get to work*,* even stretching while you’re at work*,* and then once you’re into the job your muscles are nice and warmed up.*

Training (50%) and individual knowledge (37.5%) were also mentioned as factors influencing injury risk. Instructors noted that a lack of training or knowledge increases injury risk, which is often seen in apprentices starting the trade:

*It really depends*,* I think on what you do*,* how that organization and your training. If you are properly trained*,* then I don’t think [apprentices are more at risk to get injured].*

*With no knowledge coming in*,* I guess*,* in fairness*,* you do stand a greater risk [to get injured]. Because you really don’t know what you’re going up against. When you see individuals*,* who are seasoned and what they do picking up these items that you have no idea what the weight is and just frankly maneuvering them with what seems like ease at times. I don’t think it really kind of shows you what you’re dealing with or the severity of the injuries that you can go up against.*

Awareness and attentiveness (37.5%) while working in the trade was also mentioned as a factor influencing injury susceptibility. Instructors noted that they had seen cases where a lack of awareness or attentiveness, especially in apprentices, lead to injuries:

*You can literally tell them no you should bend your knees*,* keep your back straight*,* do this and then you turn your back*,* and you can hear someone scream in pain almost*,* because they just didn’t pay any attention.*

On the other hand, they mentioned that their own awareness was something that helped them avoid injuries over the years:

*I’m just aware of it*,* I guess. and I attempt to put myself–not put myself*,* I should say*,* in potentially detrimental positions.*

The equipment used was also mentioned by two masons (25%) regarding injury risk and prevention strategies. One mason mentioned that he kept a back brace on hand, just in case as a preventative measure, while another mentioned that his trowel type contributed to a wrist injury:

*So*,* at first*,* I had the trowel*,* according to my foreman at the time*,* that put more pressure on my wrist. I switched and coincidentally*,* when I switched to the other brand*,* and the size*,* mind you*,* then my wrist injury sort of went away.*

Lastly, masons felt that susceptibility to injury depends on individual circumstance, given the number of different factors that might influence them (25%). For example, some apprentices starting the trade may have a lack of experience, but more knowledge about proper movement strategies from previous experiences as an athlete or genuine interest.

## Discussion

Masons are at high risk for MSDs and often face involuntary early retirement due to repetitive strain, heavy lifting, and awkward postures. To identify attitudes, needs, and opportunities for training masonry apprentices, we collected and analyzed the perspectives of experts in this field. Through qualitative interviews with eight instructors, each with more than 20 years of masonry work experience, and subsequent thematic analysis, we identified six themes and associated subthemes: physical demands, injuries, safety culture, learning experiences, the role of safety, and risk modifiers.

### Physical demands and MSD risk

The physical demands and MSD risks noted by instructors align with those frequently identified in the literature. Specifically, manual lifting of blocks and bricks, essential to masonry work, involves frequent and deep bending of the trunk, hips, and knees handle heavy loads [5, 61]. Consequently, masons reported severe low back injury rate of 22 per full-time equivalent worker, compared to 16.2 across all industries [62], and also exhibit a high incidence of upper extremity injuries [3, 4]. Despite a decrease in overexertion injuries to 33.4 per 10,000 full-time equivalent workers in 2015, brick and stone masons still had the third highest combined injury rate between 2015 and 2017 [63].”

In addition to the forceful lifting hazards of masonry blocks and bricks, repetitive motions were also a major concern for many of the instructors. When union and contractor representatives within the construction industry were asked about different ergonomic prevention strategies, one of the more consistent replies was increasing awareness of repetitive strain hazards and injuries [64]. Not only is repetition recognized by instructors as a hazard, but it is also important to amplify its consequences to apprentices when they are starting out in the trade.

A study on the prevalence of MSDs in masonry apprentices revealed that 78% of apprentices had symptoms associated with MSDs and these rates were similar to that of journeypersons [8]. Furthermore, this trend in MSDs was also found in other construction trades with apprentice floor layers reporting MSD symptoms at nearly the same frequency as more experienced workers [65]. This highlights that within the construction trades, and in masonry in particular, the problem of musculoskeletal pain starts early in the apprenticeship phase and extends throughout their career.

### Impact of physical demands

The high rates of injuries in masonry and the construction industry are reflected in the personal experiences of the instructors. Despite the small sample size, several instructors reported injuries due to work demands. Previous studies have found that following MSD injuries, workers in the construction trade are less likely to return to work [64]. However, all the instructors interviewed, all having returned to work post-injury, sought some form of healthcare professional to help during the recovery process or did a form of self-rehabilitation. While some had to explore several different healthcare options, those that had a greater focus on the musculoskeletal system were more helpful, such as physiotherapists or osteopaths as opposed to general practitioners. In the experiences of the instructors interviewed, either changing equipment or proper rehabilitation was critical in allowing them to return to work and resume their duties injury free. The use of healthcare practitioners, especially those focusing on musculoskeletal rehabilitation, should be encouraged post-injury to aid the return-to-work process.

### Safety culture and attitudes

#### Productivity

Productivity is a major driver of the industry, and this influence is seen in the safety culture. This is due to the pay rates of masons being based upon production, which was highlighted in the interview answers. Similarly, this view has been acknowledged in previous interviews of construction workers as one of the main obstacles to MSD prevention in the industry [64]. This underscores the need to discuss ergonomics within the framework of productivity, not only at the level of management and the return on investment but also at the level of the individual workers, who feel job pressures.

#### Injuries and role of industry

The culture and attitudes around safety in masonry were in line with other perspectives found in the literature from stakeholders in construction. For example, the instructors highlighted a belief in the trade that injuries are part of the job. This belief was also found to be held by other workers in the construction sector [64]. Furthermore, workers felt that their employers were not as committed to increasing workplace safety [64]. While this was not a theme in the interviews with the instructors, one instructor did have a strong sense of skepticism and cynicism when it came to the industry’s role in improving safety. And instructors felt that the industry had a role in setting the expectations and contributing to the overall safety culture in the trade. In one study, organizational culture was purported to have a greater effect on construction workers’ perceptions of risk than employer actions, and that construction workers were more likely to have little concern for the risks compared to other industries [66]. Instructors also felt that the training center for apprentices could do more to promote and teach ergonomics. Management commitment is one of the main barriers to ergonomic improvements [67, 68]. To improve the confidence of their workers, companies should demonstrate a commitment to employee health and safety, through time, resources, and other initiatives.

#### Socialization into the industry

Other attitudes within construction such as personal pride, the drive to gain respect from their peers or the ‘young and invincible’ belief also contributes to the overall safety culture within masonry. Similarly, the pervasiveness of ‘macho’ attitudes in the construction industry has been mentioned as one of the barriers to cultivating a strong safety culture [64]. In construction trades, apprentices “are socialized into the workforce by their mentors and other older workers” leading many to adopt and reproduce the culture, attitudes, and habits of the older generations [65]. This can often lead to resistance to change, which is well documented within the construction industry, and poses a challenge to ergonomic interventions [64, 65, 68, 69]. Resistance to change was touched upon briefly in the results with respect to resisting change to learned techniques. The influence of older workers and mentors on the apprentice during these formative years was highlighted by one instructor:

*If you’re getting into the trade*,* and you’re already starting to do things the way Papa bear did it*,* and he did it the wrong way… well then guess what? He’s setting you up for doing it wrong all your career.*

Masonry instructors mentioned the importance of training apprentices early on in their careers during their skills training courses. This is also supported by other researchers who suggest that younger workers will be more open to learning new approaches [64]. To circumvent the challenges associated with resistance to change and pre-established beliefs and habits, ergonomics training should be targeting younger workers during their formative years before they are fully socialized into the trade.

#### Shift in attitudes

Despite beliefs that ergonomics within the industry and the training center could improve, instructors highlighted positive changes in the industry over the last decade. This belief was also held within the construction industry as well [64]. This may signify that despite existing attitudes in the industry, the overall culture is shifting towards a more positive emphasis on safety and ergonomics. In the interview results, while many instructors noted that the ‘young and invincible’ attitude was ubiquitous amongst the younger workers, one instructor said that the incoming generations are much more concerned about ergonomics and safety and that it was one of the most frequently asked questions in their outreach programs. This demonstrates that despite continuing attitudes in the trade, overall, there may be a slow shift towards increased attention towards safety with the incoming generations. Boatman et al. (2015) [64] noted that the improvements in technology and tools as well as increased focus on training and awareness is leading the cultural shift.

### Education and training

Training and knowledge were identified by instructors as factors influencing injury risk in apprentices. Lack of awareness, knowledge or training about MSDs and ergonomics are also critical issues affecting MSD prevention in the construction industry [2, 64, 65, 68–71]. These include a lack of awareness of the significance of MSDs in the industry, an understanding of MSD risks and long-term consequences and costs, and basic ergonomics principles. In the U.S., 44% of Hispanic construction workers reported no formal safety training despite 67% indicating a high interest in safety training [72]. Ergonomics training is often lacking in apprenticeship programs as well [2]. This perception was also shared by the masonry instructors in their interviews about the inclusion of ergonomics information in their curriculum. Researchers agree that ergonomics training should be included at the apprenticeship stage [2, 65]. Jensen and Kofoed (2002) [65] emphasized that experienced individuals in the trade should disseminate the knowledge in order to break down resistance to change resulting from the socialization of apprentices into the workforce. The use of experienced individuals allows them as instructors to approach the apprentices with a level of credibility and respect, speak from experience, and communicate using the language of the trade. This sentiment was also shared by the instructors in the second half of the interview. Training should be practical and tailored to the context of the trade, include awareness and refresher training and apprenticeship training should be reinforced on the jobsite [69, 70, 73, 74]. For masonry training, Entzel et al. (2007) [69] suggested the inclusion of information on the job site layout, ergonomic tool use, mortar spreading technique, lifting, and adjustment of mast climbing work platforms and adjustable tower scaffolding. From a behavioral change perspective, interventions in construction should be tailored to the audience’s particular stage in the stages of change model for the highest probability of effectuating individual behavioral change, with information about MSD risks provided in the precontemplation stage, further education about MSDs and awareness of action items provided in the contemplation and preparation stage, and practical training, skills, and advice provided in the action stage [75].

### Knowledge sharing

Knowledge sharing between coworkers and from the older generation to the younger generation is critical to the learning experiences of masons and this transfer of knowledge is highlighted in the instructors’ experiences regarding MSD prevention techniques. In the literature, the social interactionist model of knowledge transfer suggests that not only is knowledge social but that it is formed within a social context [71]. This theory reinforces the importance of peer-to-peer communication as a method of knowledge sharing within the industry. To further emphasize the role of social communication, the lack of a forum to share knowledge about interventions was found as a barrier to the promotion of ergonomics within construction [67, 71]. A study on the adoption of an ergonomic intervention spread through knowledge transfer and exchange found that the use of opinion leaders (credible and connected individuals) within the construction industry could promote adoption of ergonomic interventions [71]. The practical, onsite experience of the opinion leader was valued by their peers when it came to judging the adoption of the intervention. The study showed that ergonomic change could be driven in part by a select number of key leaders and word of mouth. This mimicked the natural process of knowledge sharing that the masonry instructors highlighted in their interviews, as one of the primary ways of learning about risk prevention strategies besides experience.

### Risk modifiers and safety behaviors

Previous studies have reported that to prevent MSDs, construction workers engage in “exercising and conditioning, getting better tools and adapting tools to make them easier to use, wearing braces and pads, self-medicating with Motrin or Advil, attempting to rotate tasks or switch hands, and getting educated and talking with others” [64]. Tool, material and working technique changes are also advocated as a method to reduce MSD risk by Jensen and Kofoed (2002) [65]. All these safety behaviors and strategies were also reflected in the interviews with the masonry instructors as ways to manage the physical demands and MSD risk.

### Insights, recommendations and practical implementations

Based on the results and analysis of the interview findings, the insights and corresponding recommendations regarding ergonomic training opportunities are summarized in Table 2. Each row presents an insight derived from the interview data, alongside specific recommendations and practical steps for implementation. This tabular format highlights the connection between observed challenges and actionable interventions, making the recommendations more accessible for training program design and for informing future ergonomic initiatives in the masonry trade.

**Table 2:** Insights with corresponding recommendations and practical implementations regarding ergonomic training in masonry

Insights	Recommendations and Practical Implementations
• Physical demands in masonry contribute to higher MSD risk and general wear and tear on the body.	• Incorporate training modules on physical demands and ergonomics using real-life case studies. • Conduct regular ergonomic risk assessments to identify early MSD signs.
• Injury risk increases as masons age due to reduced strength capacity.	• Develop interventions for older masons (strength training, modified work practices).
• Many masons sustain injuries during their careers; post-injury support is important.	• Partner with healthcare providers for on-site ergonomic assessments. • Provide structured rehabilitation programs with gradual return-to-work plans.
• Injuries can be prevented through good safety behaviors.	• Deliver comprehensive safety training that emphasizes ergonomic risks.
• Belief that injuries are “part of the job” is changing with technology and safeguards.	• Reinforce leadership roles in promoting safety culture. • Develop formal ergonomics programs with training, regular assessments, and continuous improvement.
• Young masons often feel “invincible,” underestimating long-term risks.	• Promote peer education where experienced masons share injury stories and advice.
• Apprentices may lift past capacity due to pride, desire to impress, respect for leaders, laxity, or productivity pressure.	• Train apprentices to recognize and avoid these pitfalls. • Stress the importance of knowing one’s limits and balancing productivity with safety.
• Reduced productivity results from exceeding capacity or injury.	• Conduct workshops linking ergonomics to productivity. • Use data to demonstrate the long-term benefits of safe practices.
• Ergonomic discussions among masons are valuable.	• Hold regular safety meetings focused on ergonomics. • Establish communication channels for workers to raise concerns.
• Expert masons play a key role in knowledge transfer.	• Create formal mentorship programs pairing older and younger workers. • Organize sessions where expert masons share lessons learned from injuries.
• Improved fitness, stretching, technique, knowledge, equipment, and awareness reduce injury risk.	• Integrate fitness and ergonomic training into apprenticeship curricula. • Encourage warm-up/stretch routines before work. • Include modules on ergonomic tools and proper techniques.

This study has several limitations. Firstly, it was based on interviews with eight masonry instructors from a single training center, limiting the generalizability of the findings. Although these instructors had extensive experience, the small sample size reduces the robustness of the results. Future research could benefit from a larger and more diverse sample. All participants were recruited through the CMDC, which may have introduced selection bias. The instructors who participated might have had a particular interest in safety and ergonomics, potentially leading to more positive attitudes and experiences regarding safety practices. Furthermore, the presence of CMDC stakeholders during the first interview might have influenced the responses, potentially causing the interviewee to provide more favorable answers or align their responses with perceived expectations. To mitigate these biases in future work, employing random sampling techniques to select participants should be considered to ensure a more representative sample. It is also important to conduct interviews without the presence of stakeholders, who may have influenced responses. Qualitative interviews are inherently subjective and rely on self-reported information, introducing the possibility of recall bias. Participants may not accurately remember or may selectively report their experiences and attitudes. Future studies should consider incorporating quantitative data collection methods, such as surveys and questionnaires, to complement the qualitative findings and provide a more comprehensive understanding of the issues.

## Conclusion

This study found that masonry instructors emphasized the high physical demands of the trade, the variability in injury experiences, and the influence of safety culture, age, and productivity pressures on musculoskeletal health. Instructors reported that younger apprentices often seemed to view themselves as invincible and less concerned with musculoskeletal safety, while older masons were described as more aware of risks and safety requirements. A lack of formal ergonomics training was also highlighted, with instructors strongly supporting the integration of structured modules on safe lifting, injury prevention, and ergonomic awareness into apprenticeship programs. Mentorship, peer learning, and conditioning strategies were identified as additional opportunities to reduce MSD risk. These findings provide a valuable foundation for informing the design of future ergonomic training systems, which is crucial for tailoring safety interventions and apprenticeship curricula that enhance both safety and productivity in the masonry trade. Future research should evaluate the effectiveness of integrating ergonomics-focused training into apprenticeship programs and explore scalable interventions that address both safety and productivity needs.

## Data Availability

Data can be made available upon request to the corresponding author.
